# Morroniside attenuates high glucose–induced BMSC dysfunction by regulating the Glo1/AGE/RAGE axis

**DOI:** 10.1111/cpr.12866

**Published:** 2020-07-09

**Authors:** Yi Sun, Yu Zhu, Xuanzhe Liu, Yimin Chai, Jia Xu

**Affiliations:** ^1^ Department of Orthopedic Surgery Shanghai Jiao Tong University Affiliated Sixth People's Hospital Shanghai China

**Keywords:** AGE‐RAGE signalling, bone marrow mesenchymal stem cells, diabetic bone loss, glyoxalase‐1, morroniside

## Abstract

**Objectives:**

High glucose (HG)–mediated bone marrow mesenchymal stem cell (BMSC) dysfunction plays a key role in impaired bone formation induced by type 1 diabetes mellitus (T1DM). Morroniside is an iridoid glycoside derived from the Chinese herb *Cornus officinalis*, and it has abundant biological activities associated with cell metabolism and tissue regeneration. However, the effects and underlying mechanisms of morroniside on HG‐induced BMSC dysfunction remain poorly understood.

**Materials and methods:**

Alkaline phosphatase (ALP) staining, ALP activity and Alizarin Red staining were performed to assess the osteogenesis of BMSCs. Quantitative real‐time PCR and Western blot (WB) were used to investigate the osteo‐specific markers, receptor for advanced glycation end product (RAGE) signalling and glyoxalase‐1 (Glo1). Additionally, a T1DM rat model was used to assess the protective effect of morroniside in vivo.

**Results:**

Morroniside treatment reverses the HG‐impaired osteogenic differentiation of BMSCs in vitro. Morroniside suppressed advanced glycation end product (AGEs) formation and RAGE expression by triggering Glo1. Moreover, the enhanced osteogenesis due to morroniside treatment was partially blocked by the Glo1 inhibitor, BBGCP2. Furthermore, in vivo, morroniside attenuated bone loss and improved bone microarchitecture accompanied by Glo1 upregulation and RAGE downregulation.

**Conclusions:**

These findings suggest that morroniside attenuates HG‐mediated BMSC dysfunction partly through the inhibition of AGE‐RAGE signalling and activation of Glo1 and may be a potential treatment for diabetic osteoporosis.

## Graphical abstract

Under pathological diabetic conditions, chronic hyperglycaemia leads to the increased accumulation of advanced glycation end products (AGEs) by methylglyoxal (MG) and activation of receptor for advanced glycation end product (RAGE) signalling. Subsequently, AGE‐RAGE activation induces bone marrow mesenchymal stem cell (BMSC) dysfunction due to a combination of decreased osteogenic differentiation of BMSCs and enhanced inflammation, reactive oxygen species generation and BMSC apoptosis. Finally, reduced osteogenesis of BMSCs causes impaired bone formation in type 1 diabetes. Morroniside triggers Glo1 to degrade MG, thus downregulating AGE‐RAGE signalling, rectifying these downstream detrimental effects and attenuating BMSC dysfunction induced by high glucose.

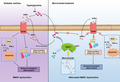

## 1 INTRODUCTION

Diabetic osteoporosis is an increasingly underrecognized complication of type 1 diabetes mellitus (T1DM), which is characterized by low bone mass and microarchitectural alterations.^1^ Compared with the risks to non‐diabetic patients, individuals with T1DM have a 2‐fold to 7‐fold increased risk of hip fracture and a 2‐fold to 5‐fold increased risk of spinal fracture.^2^ Compared with post‐menopausal osteoporosis, T1DM‐related bone loss is primarily mechanistically characterized by impaired bone formation instead of enhanced bone resorption.^3^ This impaired bone formation is mainly attributed to T1DM‐related chronic hyperglycaemia disturbing the functions of bone marrow mesenchymal stem cells (BMSCs), which are the precursors of osteoblasts.^4^, ^5^, ^6^, ^7^ Therefore, an urgent need remains for novel, reparative therapeutic strategies that target high glucose (HG)–induced BMSC dysfunction to prevent diabetic bone loss and fragility.

Advanced glycation end products (AGEs) are formed by non‐enzymatic reactions under chronic hyperglycaemia.^8^ AGEs can interact with receptor for advanced glycation end products (RAGE) and contribute to HG‐induced cell deterioration and numerous diabetic complications.^9^ In the skeleton, it has been increasingly recognized that pathological activation of AGE‐RAGE signalling inhibits the osteogenic differentiation of BMSCs.^10^, ^11^, ^12^ Moreover, activation of RAGE also enhances apoptosis, inflammation and oxidative stress, which subsequently reduce the viability and function of BMSCs and amplify HG‐induced BMSC dysfunction.^13^ These findings suggest that RAGE may be a promising target for treating HG‐induced BMSC dysfunction.

AGE‐RAGE signalling is mainly regulated by the glyoxalase system as an upstream factor, and glyoxalase‐1 (Glo1) is the rate‐limiting enzyme. Physiologically, Glo1 degrades the major precursor of AGEs, namely methylglyoxal (MG), with glutathione (GSH) as a cofactor.^14^ However, under chronic hyperglycaemia, both the function and expression of Glo1 and GSH are reduced, which exacerbates the accumulation of AGEs and the pathological activation of AGE‐RAGE signalling.^15^, ^16^ Therefore, enhancing the expression and function of Glo1 exhibits great therapeutic potential for treating RAGE‐mediated BMSC dysfunction.

*Cornus officinalis*, a traditional Chinese herbal medicine in East Asia, is widely used as the main component of many famous herbal drugs for treating bone diseases and diabetes.^17^, ^18^, ^19^ Morroniside is considered the main active component of *C officinalis*, and it shows abundant biological activities, including anti‐apoptosis,^20^ antioxidative stress^21^, ^22^ and anti‐ischaemic effects.^23^ Moreover, morroniside has been reported to regulate RAGE signalling and prevent the damaging effect of AGEs in diabetic nephropathy.^24^ However, the protective effect of morroniside on diabetic‐related bone loss and BMSC dysfunction has not been reported thus far.

Herein, we observed that morroniside restores the osteogenic differentiation of rat BMSCs exposed to high‐glucose conditions, which occurs partly via the inhibition of AGE‐RAGE signalling through the activation of Glo1. Moreover, the effect of morroniside on bone tissue was assessed in a rat type 1 diabetic osteoporosis model, and the results showed that morroniside attenuated the bone mass loss and improved the bone microarchitecture. Based on our findings, morroniside can attenuate HG‐induced BMSC dysfunction and relieve impaired bone formation in patients with T1DM.

## 2 MATERIALS AND METHODS

### 2.1 Isolation and culture of rat BMSCs

The isolation and culture of rat BMSCs followed our previously published protocol.^25^ Briefly, 4‐week‐old Sprague Dawley rats were euthanized and sterilized. Then, femoral bone marrow was flushed with low‐glucose (LG; 5.5 mmol/L) Dulbecco's modified Eagle's medium (DMEM; HyClone, PA, USA) and centrifuged at 1000 rpm for 5 minutes; the cell pellets were resuspended and cultured in LG‐DMEM supplemented with 10% foetal bovine serum (FBS; Gibco, NY, USA) and 1% penicillin/streptomycin (Gibco) at 37 ℃ with 5% CO_2_.

To prepare the BMSCs for the HG treatment, after the first passage, the cells were serum starved for 24 hours in LG‐DMEM with 5% FBS. Then, the cells were cultured in HG (25 mmol/L) DMEM for two weeks and then harvested for further analysis.^26^ Morroniside was dissolved in phosphate‐buffered saline (PBS) at different concentrations (1, 10 and 100 μmol/L) for dose‐dependent treatments except for the Counting Kit‐8 (CCK‐8) assay, where we added a group of 1000 μmol/L morroniside. When the concentration of morroniside is not specifically defined, the concentration was 100 μmol/L. 4‐chloro‐N‐cyclohexyl‐N‐(phenylmethyl)‐benzamide (FPS‐ZM1; Sigma‐Aldrich, MO, USA) and S‐p‐bromobenzylglutathione cyclopentyl diester (BBGCP_2_; Sigma‐Aldrich) was dissolved in dimethyl sulfoxide (DMSO; Sigma‐Aldrich) and further diluted to 10 μmol/L and 15 μmol/L, respectively, with culture medium.^12^, ^27^ Cells from passages 4 to 6 were used in the experiments.

### 2.2 Cell viability assay

The effect of morroniside and HG on BMSC proliferation was assessed via the CCK‐8 assay (Dojindo, Kyushu Island, Japan) following the method described in our previous study.^25^ In brief, BMSCs were seeded into 96‐well plates at a density of 2000 cells/well with different concentrations of morroniside (0, 1, 10, 100 and 1000 μmol/L) or glucose (5.5, 10, 25 and 40 mmol/L) and the CCK‐8 assay was performed continuously for 6 days according to the manufacturer's instructions. A microplate (Thermo, MA, USA) was used to measure the absorbance at 450 nm.

### 2.3 Osteogenic differentiation protocol

The protocol for BMSC osteogenic differentiation was described in our previous study.^25^ Briefly, HG‐treated BMSCs were seeded into 24‐well or 6‐well plates at a density of 1 × 10^4^ cells/cm^2^ and then cultured in HG‐DMEM (25 mmol/L), and BMSCs cultured in LG‐DMEM (5.5 mmol/L) were considered the control group. After the cells reached 80% confluence, the medium was replaced with osteogenic induction medium (OIM), and 1 nmol/L dexamethasone, 50 μmol/L L‐ascorbic acid‐2‐phosphate and 20 mmol/L β‐glycerophosphate (Cyagen Biosciences, Guangzhou, China) were added to the corresponding culture medium with different glucose concentrations. Morroniside, FPS‐ZM1 and BBGCP_2_ were added to the OIM with the concentrations mentioned above.

### 2.4 ALP staining and ALP activity measurement

After culturing in OIM for 3 days, alkaline phosphatase (ALP) staining and ALP activity assays were performed. For ALP staining, BMSCs were washed with PBS three times and fixed with 4% paraformaldehyde for 15 minutes. After washing, the cells were stained using BCIP/NBT ALP Color Development Kit (Beyotime, Shanghai, China) according to the manufacturer's instructions. To assessing the ALP activity, BMSCs were lysed by a radioimmunoprecipitation assay (RIPA) lysis buffer (Sigma‐Aldrich) and then analysed using an Alkaline Phosphatase Assay Kit (Beyotime) following the manufacturer's instructions. The OD value was measured at 520 nm.

### 2.5 Alizarin red staining

After 14 days of osteogenic induction, the cells were fixed with 4% paraformaldehyde for 15 minutes, washed with PBS three times and then stained with Alizarin red (Cyagen Biosciences) for 5 minutes. To quantify the staining, stained mineralized nodules were desorbed with 10% cetylpyridinium chloride (Sigma‐Aldrich) and the OD value was measured at 570 nm.

### 2.6 RNA extraction and quantitative reverse transcription polymerase chain reaction (qRT‐PCR)

Total RNA was extracted using TRIzol reagent (Ambion, New York, USA). cDNA was synthesized using the PrimeScript RT Master Mix (Takara Bio, Otsu, Japan). qRT‐PCR was performed using the SYBR Green I Master Mix (EZBioscience, Roseville, USA) following the manufacturer's instructions on an ABI 7900 system (Applied Biosystems, USA). Primers were provided by BioTNT (BioTNT, Shanghai, China), and the sequences were as follows: runt‐related transcription factor 2 (*Runx2*) forward: 5′ ACTTCCTGTGCTCGGTGCT 3′, reverse: 5′ GACGGTTATGGTCAAGGTGAA 3′; *Col1* forward: 5′ CATCGGTGGTACTAAC 3′, reverse: 5′ CTGGATCATATTGCACA 3′; osteopontin (*Opn*) forward: 5′ ATCTCCTAGCCCCACAGACC 3′, reverse: 5′ TCCGTGGGAAAATCAGTGACC 3′; osteocalcin (*Ocn*) forward: 5′ CCTCACACTCCTCGCCCTATT 3′, reverse: 5′ CCCTCCTGCTTGGACACAAA 3′; *Alp* forward: 5′ ACCATTCCCACGTCTTCACATTT 3′, reverse: 5′ AGACATTCTCTCGTTCACCGCC 3′; bone morphogenetic protein‐2 (*Bmp2*) forward: 5′ ACTCGAAATTCCCCGTGACC 3′, reverse: 5′ CCACTTCCACCACGAATCCA 3′; *Rage* forward: 5′ ACAGAAACCGGTGATGAAGG 3′, reverse: 5′ ATTCAGCTCTGCACGTTCCCT 3′; *Glo1* forward: 5′ CGAGGGTTCTTGGACTGACG 3′, reverse: 5′ TTCAGTGCCCCAGTTGTGTG 3′; and glyceraldehyde‐3‐phosphate dehydrogenase (*Gapdh*) forward: 5′ GGCATGGACTGTGGTCATGAG 3′, reverse: 5′ TGCACCACCAACTGTTAGC 3′. PCR was performed for 40 cycles. The expression levels of the target gene mRNA were calculated relative to that of *Gapdh* using the 2^‐ΔΔCt^ method.

### 2.7 Western blots (WB)

Cells or bone tissues were lysed in RIPA lysis buffer, centrifuged, diluted with loading buffer (5×; Sigma‐Aldrich) and then heated at 95℃ for 6 minutes. The protein concentration was determined using BCA protein assay kit (Cell Signaling Technology, MA, USA). Subsequently, protein samples were separated by 10% sodium dodecyl sulphate polyacrylamide gel electrophoresis and blotted onto polyvinylidene fluoride membranes (Millipore, Billerica, MA, USA). The membranes were blocked in 5% non‐fat milk for 2 hours and incubated in primary antibodies specific to RUNX2 (Cell Signaling Technology), COL1 (Proteintech, IL, USA), OCN (Abcam, Cambridge, UK), RAGE (Abcam), GLO1 (Abcam) and GAPDH (Cell Signaling Technology) at 4 ℃ overnight. After washing, the membranes were incubated with horseradish peroxidase–conjugated secondary antibodies (Proteintech) for one hour at room temperature. After incubation with a Femtolight Chemiluminescence kit (Epizyme, Shanghai, China), protein intensities were evaluated by an ECL Plus Western Blotting Detection System (GE Healthcare, IL, USA).

### 2.8 Enzyme‐linked immunosorbent assay (ELISA)

Intracellular AGE levels and inflammatory cytokines were assessed by ELISA kits specific to AGEs (Sigma‐Aldrich), TNF‐α, IL‐1β and IL‐6 (Neobioscience, Shenzhen, China) according to the manufacturer's instructions.

### 2.9 Glo1 activity measurement

Glo1 activity was measured using a Glyoxalase‐1 Activity Assay Kit (Sigma‐Aldrich) according to the manufacturer's instructions. The OD value was measured at 240 nm every 2 minutes. Glo1 activity was shown as the rate of change in A_240_ per mg protein.

### 2.10 GSH level measurement

The level of GSH was assessed by a GSH/GSSG Ratio Detection Kit (Nanjing Jiancheng Biotechnology Co Ltd., Jiangsu, China) following the manufacturer's instructions. The OD value was measured at 420 nm.

### 2.11 Apoptosis detection by flow cytometry

Apoptosis was assessed by an Annexin V‐FITC/PI apoptosis detection kit (Dojindo) following the manufacturer's instructions. Guava easyCyte™ Systems (Luminex, TX, USA) were used for analysis.

### 2.12 Intracellular ROS measurement

Intracellular ROS was estimated by a Reactive Oxygen Species Assay Kit (Beyotime, Shanghai, China) according to the manufacturer's instructions. Flow cytometry was used to measure the mean fluorescence intensities at Ex 488/Em 525.

### 2.13 Establishment of the rat T1DM model

All rats handling and procedures were approved by the Animal Research Committee of Shanghai Jiao Tong University Affiliated Sixth People's Hospital. Twenty 4‐week‐old male Sprague Dawley rats received intraperitoneal injections of streptozotocin (Sigma‐Aldrich; 60 mg/kg) to induce T1DM, and five rats in the control group received vehicle injections. Blood glucose concentrations were evaluated after 3 and 7 days by a blood glucose meter (Accu‐Chek; Roche Diagnostics, Indianapolis, IN, USA). If the blood glucose concentration was higher than 16.7 mmol/L, the rats were diagnosed with T1DM. Eighteen rats fit the criterion and were randomly grouped into three experimental cohorts with six rats each group: DM, DM + 15 μg/kg morroniside (DM + M15) and DM + 30 μg/kg morroniside (DM + M30). The other seven rats were grouped into the control group. Morroniside was administered by intraperitoneal injection daily for seven weeks after the establishment of T1DM. The body weights and blood glucose concentrations were monitored each week. Eight weeks after injection, the rats were sacrificed, and femurs were collected for micro‐CT and histological analysis.

### 2.14 Micro‐CT analysis

A micro‐CT scanner (Skyscan 1172; Bruker micro‐CT, Kontich, Belgium) was utilized with the following parameters: X‐ray energy: 80 kVp; current: 112 μA; and exposure time: 370 ms Distal femoral trabecular bone was selected to evaluate the bone mineral density (BMD), bone volume/total volume (BV/TV), trabecular number (Tb.N), trabecular thickness (Tb.Th), structure model index (SMI) and trabecular separation (Tb.Sp). In addition, the mid‐diaphysis was also assessed by the cortical bone area (Ct.Ar) and cortical thickness (Ct.Th). Three‐dimensional reconstruction images of the trabecular bone were obtained using CTvox 3.0 software (Bruker, German).

### 2.15 Histological and immunohistochemical analysis

After decalcification with 10% EDTA, the distal femurs were embedded in paraffin and sectioned coronally at a thickness of 5 μm. Haematoxylin and eosin (H&E) staining was performed as described in our previous study.^25^ For the immunohistochemical (IHC) analysis, we followed the methods described in our previous study.^28^ The mean IOD of staining (IOD/area) was assessed on each section from five random fields that were selected from the distal metaphyseal of the rat femur using Image‐Pro Plus 6.0 software (Media Cybernetics, MD, USA).

### 2.16 Statistical analysis

SPSS 22.0 software (IBM, NY, USA) was utilized to analyse the data, which are displayed as the mean ± standard deviation (SD). Data were compared with two‐tailed Student's *t* tests. A value of *P* < .05 was defined as significant.

## 3 RESULTS

### 3.1 Morroniside does not affect BMSC proliferation

**FIGURE 1:**
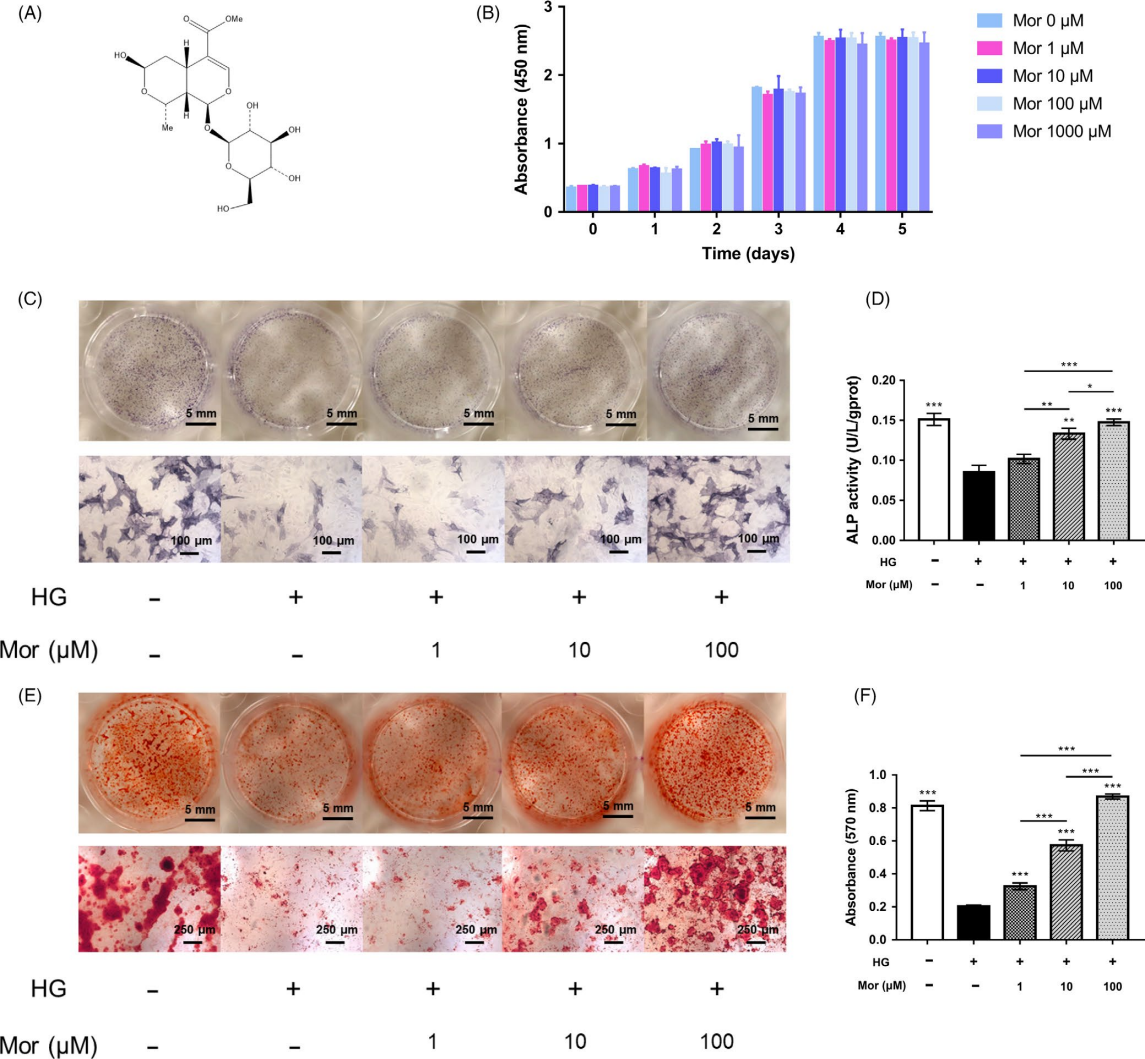
Morroniside improves the osteogenic differentiation of BMSCs exposed to high glucose in vitro. A, Chemical structure of morroniside. B, Cell viability in response to morroniside treatment was evaluated by a CCK‐8 assay. C‐F, Osteogenesis was determined by ALP staining (C), ALP activity assays (D) and Alizarin Red staining (E). Calcium deposition was assessed by measuring the optical density (F). The data were confirmed by three repeated tests and are presented as the means ± SD. Mor, morroniside. HG, high glucose. *P < .05 compared with high glucose treatment group; **P < .01; ***P < .001

Morroniside is a natural iridoid glycoside, which is an atypical secoiridoid containing a six‐membered cyclic inner ether fragment (Figure 1A). To investigate the effect of morroniside on BMSC proliferation, we performed a CCK‐8 assay. Cell proliferation was not significantly changed by treatment with morroniside at 1, 10, 100, 1000 μmol/L for 1‐5 days compared with that of the control group (*P* > .05) (Figure 1B). These results demonstrated that morroniside had no cytotoxic effects on BMSCs at these doses.

### 3.2 Morroniside reverses osteogenic differentiation of BMSCs exposed to high glucose in vitro

To investigate the effect of HG and the protective potential of morroniside on the osteogenesis of BMSCs in vitro, ALP staining, ALP activity assays and Alizarin Red staining were performed. Reduced ALP staining, ALP activity and mineralized nodule formation were observed in the HG treatment group compared to that of the normal glucose control group (Figure 1C‐F). However, this impaired osteogenic differentiation of BMSCs induced by HG treatment was partially reversed by morroniside treatment: both ALP staining and mineralized nodule formation were increased after morroniside treatment in a dose‐dependent manner relative to the untreated HG group (Figure 1C‐F).

**FIGURE 2:**
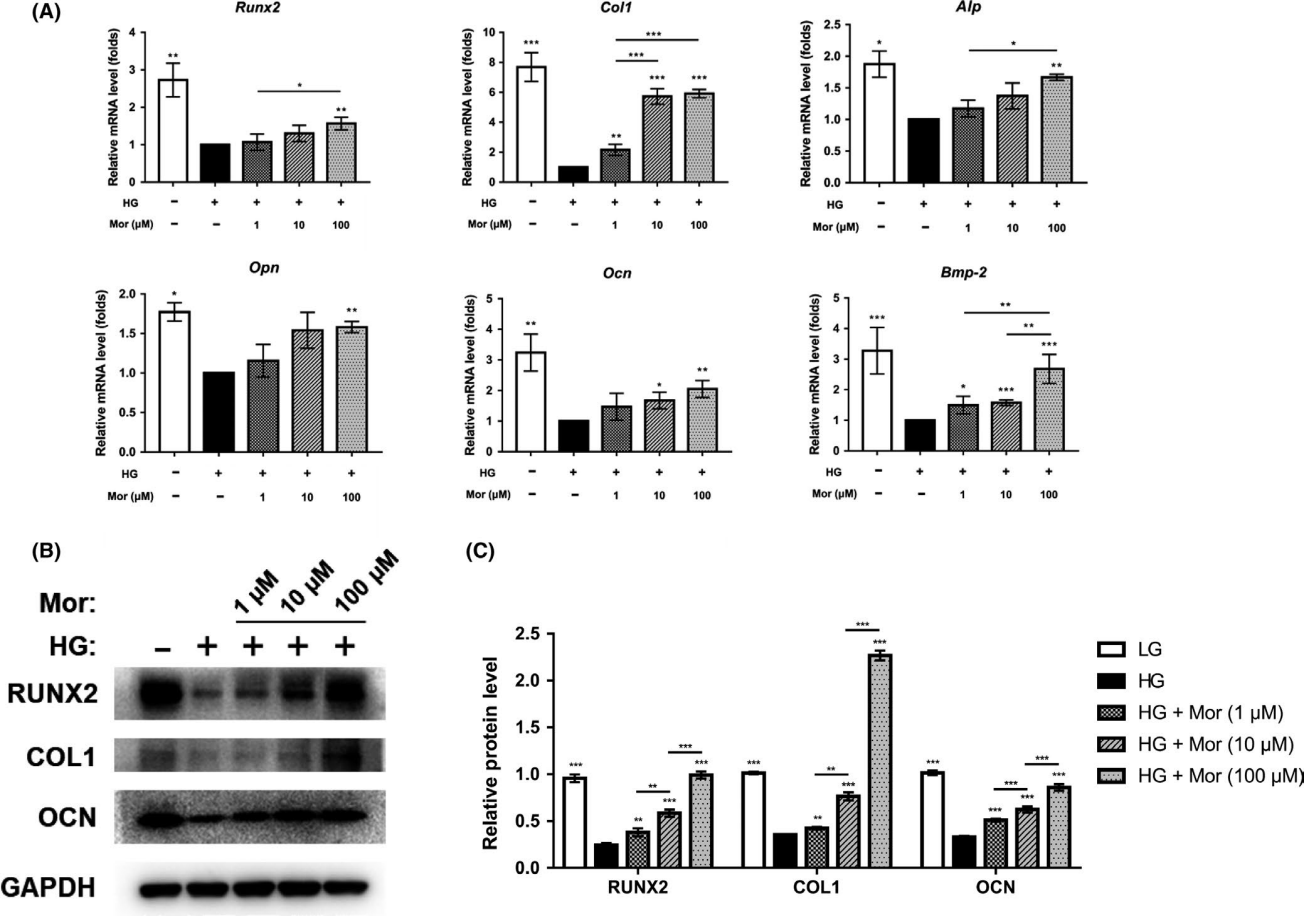
Morroniside enhances the expression of osteogenic‐specific genes and proteins that are reduced by high glucose conditions. A‐C, Expression of osteogenic‐specific genes and proteins was assessed by qRT‐PCR (A) and Western blot (B, C) at day 7 of osteogenic differentiation. The data were confirmed by three repeated tests and are presented as the means ± SD. Mor, morroniside. HG, high glucose. *P < .05 compared with high glucose treatment group; **P < .01; ***P < .001

Furthermore, the expression levels of *Runx2, Col1, Opn, Ocn, Alp* and *Bmp2*, which are osteo‐specific genes, were measured by qRT‐PCR. These mRNA levels were reduced in the HG group and restored after morroniside treatment at day 7 (Figure 2A). Consistently, the WB results showed that the protein levels of RUNX2, COL1 and OCN were decreased under HG conditions, and morroniside attenuated these damaging effects in a dose‐dependent manner (Figure 2B,C).

### 3.3 Morroniside inhibits AGE‐RAGE signalling possibly through glyoxalase 1

**FIGURE 3:**
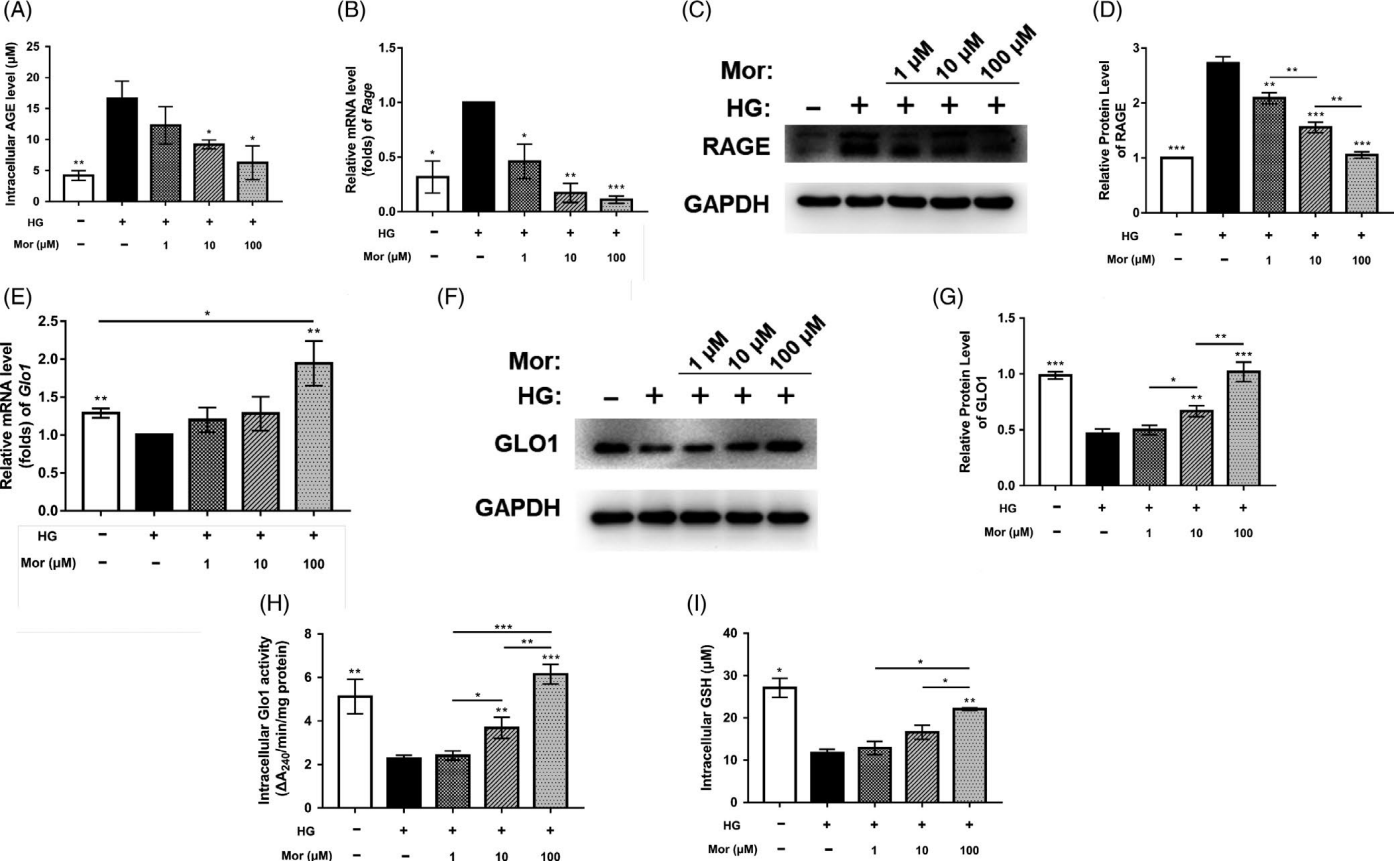
Morroniside inhibits AGE‐RAGE signalling and activates Glo1. A, Intracellular level of AGEs was evaluated by ELISA. B‐D, Gene and protein expression of RAGE was assessed by qRT‐PCR (B) and Western blot (C, D) at day 7 after treatment. E‐G, Gene and protein expression of Glo1 was determined by qRT‐PCR (E) and Western blot (F, G) at day 7. H and I, Glo1 activity was measured by Glo1 activity assays (H) and a GSH detection assay (I). The data were confirmed by three repeated tests and are presented as the means ± SD. Mor, morroniside. HG, high glucose. *P < .05 compared with high glucose treatment group; **P < .01; ***P < .001

The RAGE signalling pathway has been reported to be involved in HG‐induced BMSC dysfunction. To further determine the detailed mechanism of the protective effect of morroniside, we measured the expression of AGE‐RAGE signalling‐related genes and proteins. The ELISA results showed that AGE level in the HG group was significantly increased compared with that in the control group; however, morroniside treatment significantly reduced the AGE level in BMSCs (*P* < .05) (Figure 3A). Similarly, the qRT‐PCR and WB results demonstrated that the mRNA and protein levels of RAGE were both enhanced by HG treatment (*P* < .05, *P* < .001) and partially restored by morroniside (*P* < .001) (Figure 3B‐D).

Glo1 degrades the precursor of AGEs (MG) and further inhibits AGE‐RAGE signalling. To investigate the effect of morroniside on Glo1, we measured the expression levels of Glo1 mRNA and protein. The qRT‐PCR results showed that *Glo1* expression decreased by 22% in the HG group (*P* < .01) and was further significantly enhanced by 100 μmol/L morroniside (*P* < .01) (Figure 3E). Treatment with 1 μmol/L and 10 μmol/L morroniside had no significant effects on *Glo1* expression. The WB analysis results showed that after morroniside treatment, the expression level of GLO1 was increased in a dose‐dependent manner (Figure 3F,g). Similarly, HG also caused a decrease in intracellular Glo1 activity, whereas this activity was significantly increased by morroniside (Figure 3H). In addition, we measured the level of GSH, which is an indispensable coenzyme for Glo1 function and found that intracellular GSH was decreased in BMSCs exposed to HG, whereas this decrease was reversed after morroniside treatment (Figure 3I).

### 3.4 BBGCP_2_ (a Glo1 inhibitor) partially relieves the inhibitory effect of morroniside on AGE‐RAGE signalling

**FIGURE 4:**
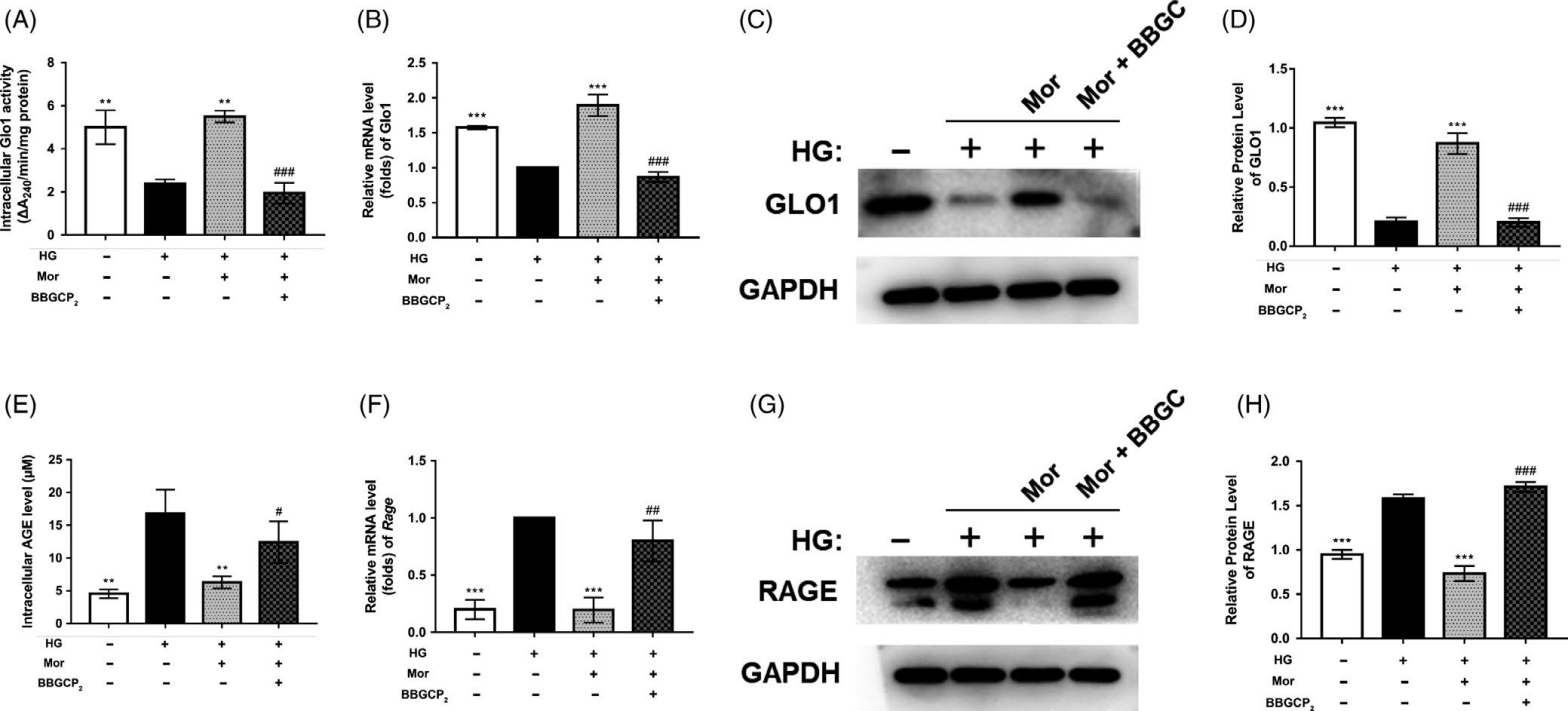
Activation of Glo1 and inhibition of AGE‐RAGE signalling by morroniside treatment are partially blocked by a Glo1 inhibitor (BBGCP_2_). A, Glo1 activity was measured by Glo1 activity assays. B‐D, Gene and protein expression of Glo1 was determined by qRT‐PCR (B) and Western blot (C, D) at day 7 after treatment. E, Intracellular level of AGEs was assessed by ELISA. F‐H, Gene and protein expression of RAGE was determined by qRT‐PCR (F) and Western blot (G, H) at day 7. The data were confirmed by three repeated tests and are presented as the means ± SD. Mor, morroniside. HG, high glucose. BBGC, BBGCP_2_. *P < .05 compared with the high glucose treatment group; **P < .01; ***P < .001. #*P* < .05 compared with the morroniside treatment group; ##*P* < .01; ###*P* < .001

To explore whether the suppression effect of morroniside on AGE‐RAGE signalling is mediated by Glo1, we cotreated BMSCs with a Glo1 inhibitor, BBGCP_2_ and morroniside. After BBGCP_2_ treatment, both the expression and the activity of Glo1 were reduced compared with that of the HG + M group (Figure 4A‐D). Furthermore, the AGE level and RAGE expression were increased in the BBGCP_2_ + HG + M group compared with the HG + M group as indicated by the ELISA, qRT‐PCR and WB results (Figure 4E‐H). These results demonstrated that the suppressive effect of morroniside on AGE‐RAGE signalling was partially reversed by Glo1 inhibition.

### 3.5 BBGCP_2_ partially inhibits the morroniside‐mediated increase in osteogenesis in HG‐treated BMSCs

**FIGURE 5:**
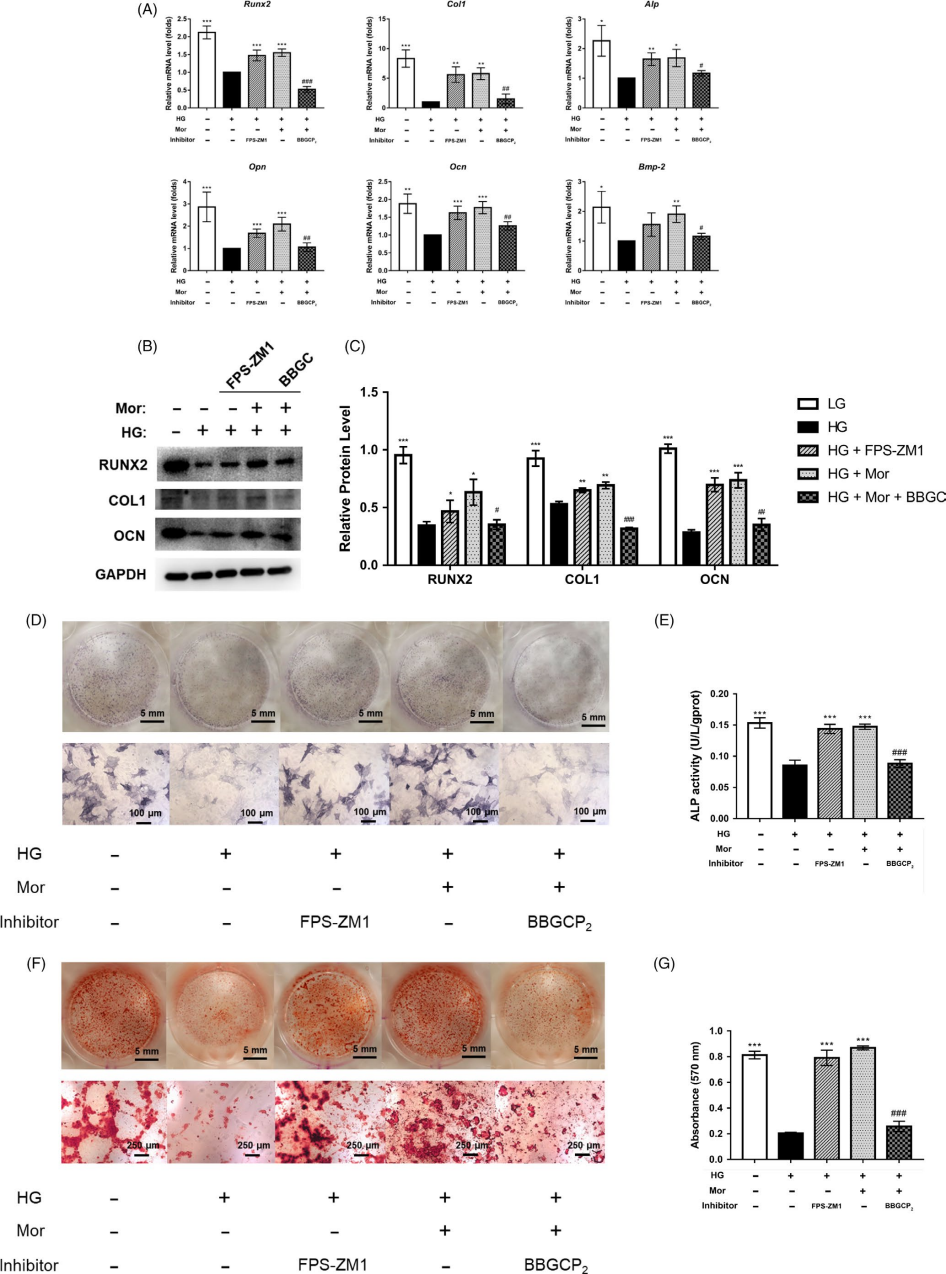
Increased osteogenesis of BMSCs induced by morroniside treatment under high glucose conditions is partially inhibited by BBGCP_2_. A‐C, Expression of osteogenic‐specific genes and proteins was assessed by qRT‐PCR (A) and Western blot (B, C) at day 7 of osteogenic differentiation. D‐G, Osteogenesis was determined by ALP staining (D), ALP activity assays (E) and Alizarin Red staining (F). Calcium deposition was assessed by measuring the optical density (G). The data were confirmed by three repeated tests and are presented as the means ± SD. Mor, morroniside. HG, high glucose. FPS‐ZM1, an inhibitor of RAGE signalling. BBGC, BBGCP_2_. *P < .05 compared with the high glucose treatment group; **P < .01; ***P < .001. #*P* < .05 compared with the morroniside treatment group; ##*P* < .01; ###*P* < .001

To further confirm the involvement of Glo1, the effect of Glo1 inhibition on osteogenesis was investigated as well. Inhibition of Glo1 directly reduced the suppressive effect of morroniside on AGE‐RAGE signalling and partially reduced the positive effect of morroniside on the expression levels of osteo‐related genes and proteins (Figure 5A‐C). In addition, ALP staining and activity assay showed that enhancement of ALP expression was reduced after adding BBGCP_2_ compared to that with morroniside treatment without BBGCP_2_ (Figure 5D,E). A similar result was observed in which BBGCP_2_ inhibited the formation of mineralized nodules, as measured by Alizarin Red staining (Figure 5F,G).

To confirm the direct effect of RAGE inhibition on HG‐induced BMSC dysfunction, a positive control, FPS‐ZM1 (an inhibitor of RAGE), was also included in this experiment. Subsequently, inhibition of RAGE enhanced the expression of osteo‐related genes and proteins that were decreased by HG treatment (Figure 5A‐C).

### 3.6 Morroniside has a protective effect on high glucose–induced apoptosis, inflammation and ROS generation in BMSCs, which was partially reduced by BBGCP_2_

**FIGURE 6:**
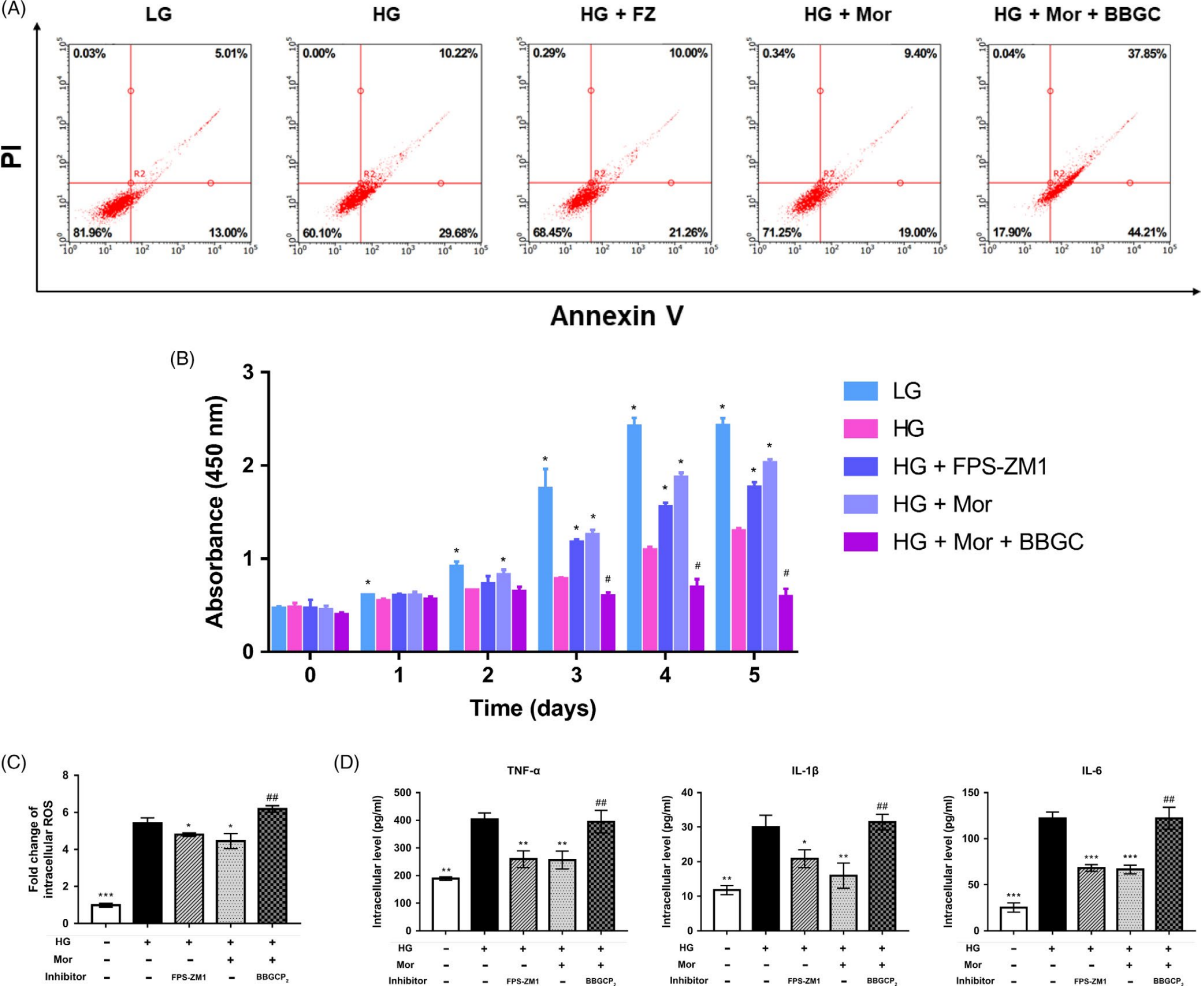
Morroniside has positive effects on apoptosis, inflammation and ROS generation promoted by high glucose in BMSCs, and these effects are partially reduced by BBGCP2. A, BMSC apoptosis was evaluated by Annexin V/PI assays on day 3 after treatment. B, Cell viability was evaluated by CCK‐8 assays. C, ROS generation in BMSCs was assessed by ROS assay kits. D, Inflammatory cytokines in BMSCs were assessed by ELISA. The data were confirmed by three repeated tests and are presented as the means ± SD. Mor, morroniside. HG, high glucose. FPS‐ZM1, an inhibitor of RAGE signalling. BBGC, BBGCP_2_. *P < .05 compared with the high glucose treatment group; **P < .01; ***P < .001. #*P* < .05 compared with the morroniside treatment group; ##*P* < .01; ###*P* < .001

To further investigate the role of morroniside on BMSCs, we evaluated apoptosis, cell proliferation inflammation and oxidative stress after treatment with morroniside. The results of flow cytometry showed that BMSC apoptosis was significantly enhanced after exposure to HG for 72 hours, compared with the control group. However, the enhanced BMSCs apoptosis under HG conditions was reduced after morroniside treatment. Furthermore, this positive effect of morroniside on BMSC apoptosis was blocked by BBGCP_2_ (Figure 6A). In addition, the CCK‐8 results also showed that the BMSC viability was significantly reduced by HG in a dose‐dependent manner (Figure S1). Moreover, morroniside restored the decreased cell viability induced by HG, and this protective effect of morroniside was also inhibited by the BBGCP_2_ treatment (Figure 6B). Furthermore, the ELISA and DHCA results indicated that the expression levels of proinflammatory mediators (including TNF‐α, IL‐1β and IL‐6) and intracellular ROS were markedly enhanced by HG and reduced by morroniside (Figure 6C). These protective effects of morroniside were also blocked by the BBGCP_2_ treatment (Figure 6C,D).

### 3.7 Morroniside enhances bone mineral density and improves bone microarchitecture in a rat type 1 diabetic model

**FIGURE 7:**
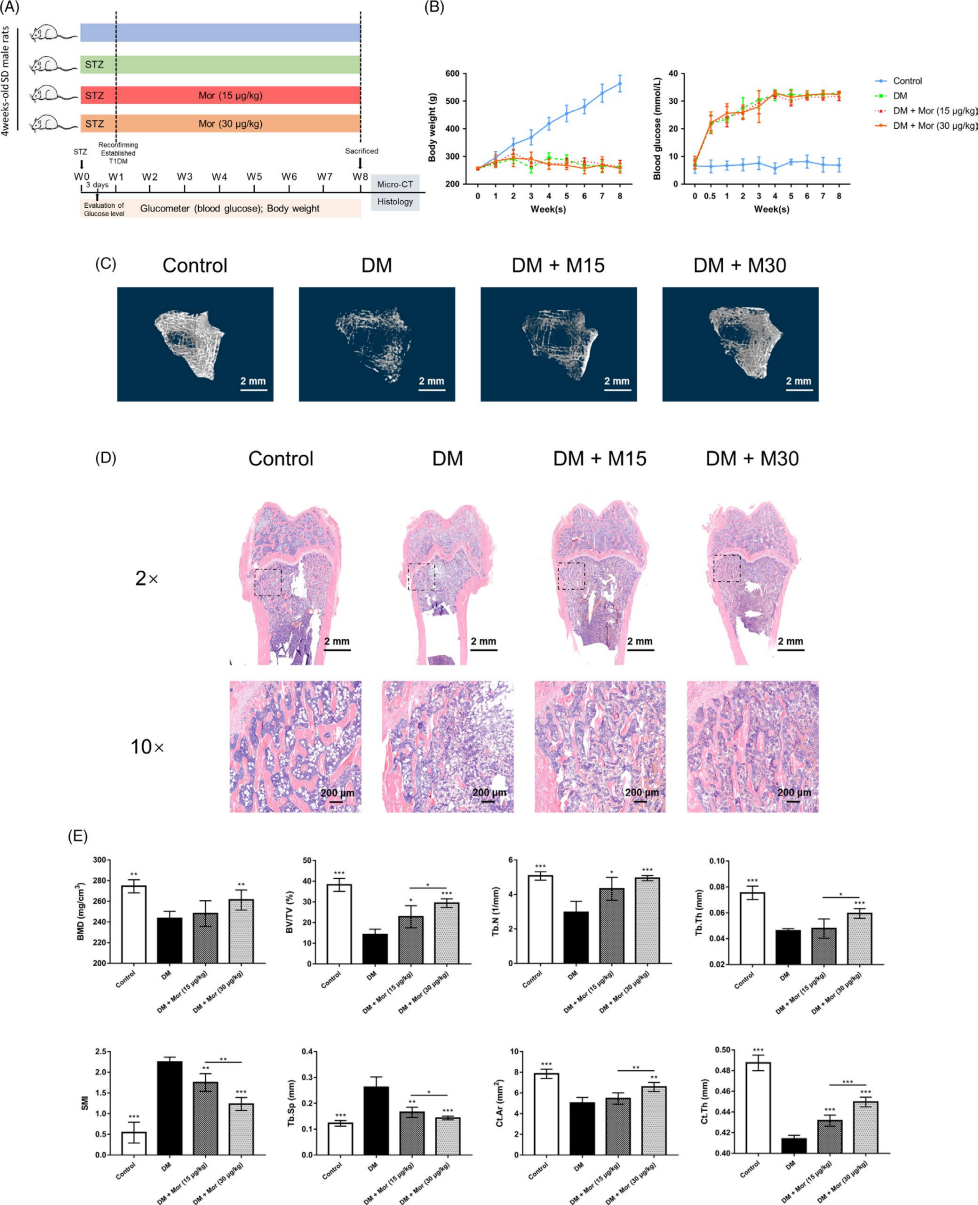
Morroniside promotes bone mineral density and improves bone microarchitecture in a rat type 1 diabetes model. A, Experimental design illustrating the time points for inducing diabetes by STZ, establishing T1DM and collecting parameters, as well as the timing and dose of morroniside administration in the animal model. B, Weight and serum glucose levels of rats during the experiment. C, Microcomputed tomography–generated three‐dimensional reconstruction images of the trabecular bone at the distal femur in each group. D, Representative images of H&E staining in sections of the rat distal femur, at magnifications of 20 × and 100×. E, Micro‐CT analyses of bone mineral density (BMD), bone volume/total volume (BV/TV), trabecular number (Tb.N), trabecular thickness (Tb.Th), structure model index (SMI), trabecular separation (Tb.Sp), cortical bone area (Ct.Ar) and cortical thickness (Ct.Th). The data were confirmed by three repeated tests and are presented as the means ± SD. Mor, morroniside. M15, 15 μg/kg morroniside treatment. M30, 30 μg/kg morroniside treatment. *P < .05 compared with the DM group; **P < .01; ***P < .001

The design of the animal experiment is shown in Figure 7A. Eighteen of the twenty rats that received the STZ injection were diabetic at 7 days after injection. All diabetic rats remained hyperglycaemic until the end of the study and exhibited lower body weights than normal rats. Morroniside treatment did not affect glycaemia or body weight (Figure 7B).

As shown in Figure 7C, a 3D reconstruction of the trabecular bone at the distal femurs clearly showed a reduction in trabecular bone mass and the breakage of cancellous bone in diabetic rats, and the administration of morroniside improved the mass and microarchitecture of the distal femur trabecular bone. Moreover, micro‐CT analysis of the distal femur showed decreased BMD in the DM group and improved BMD in the DM + M30 group but not the DM + M15 group (by 7.3%) (Figure 7E). Other parameters, including BV/TV, Tb.N, Tb.Th, Ct.Ar and Ct.Th, were significantly decreased, while SMI and Tb.Sp were significantly increased in DM group. These changes were all attenuated by the morroniside treatment (*P* < .05) (Figure 7E). Consistent with the micro‐CT analysis results, H&E staining of the distal femur sections confirmed a reduction in trabecular number and trabecular thickness in diabetic rats compared with that of the control, and these reductions were attenuated by morroniside treatment (Figure 7D).

### 3.8 Morroniside restores the expression of RAGE and osteogenic markers to the control levels in diabetic rats in vivo

**FIGURE 8:**
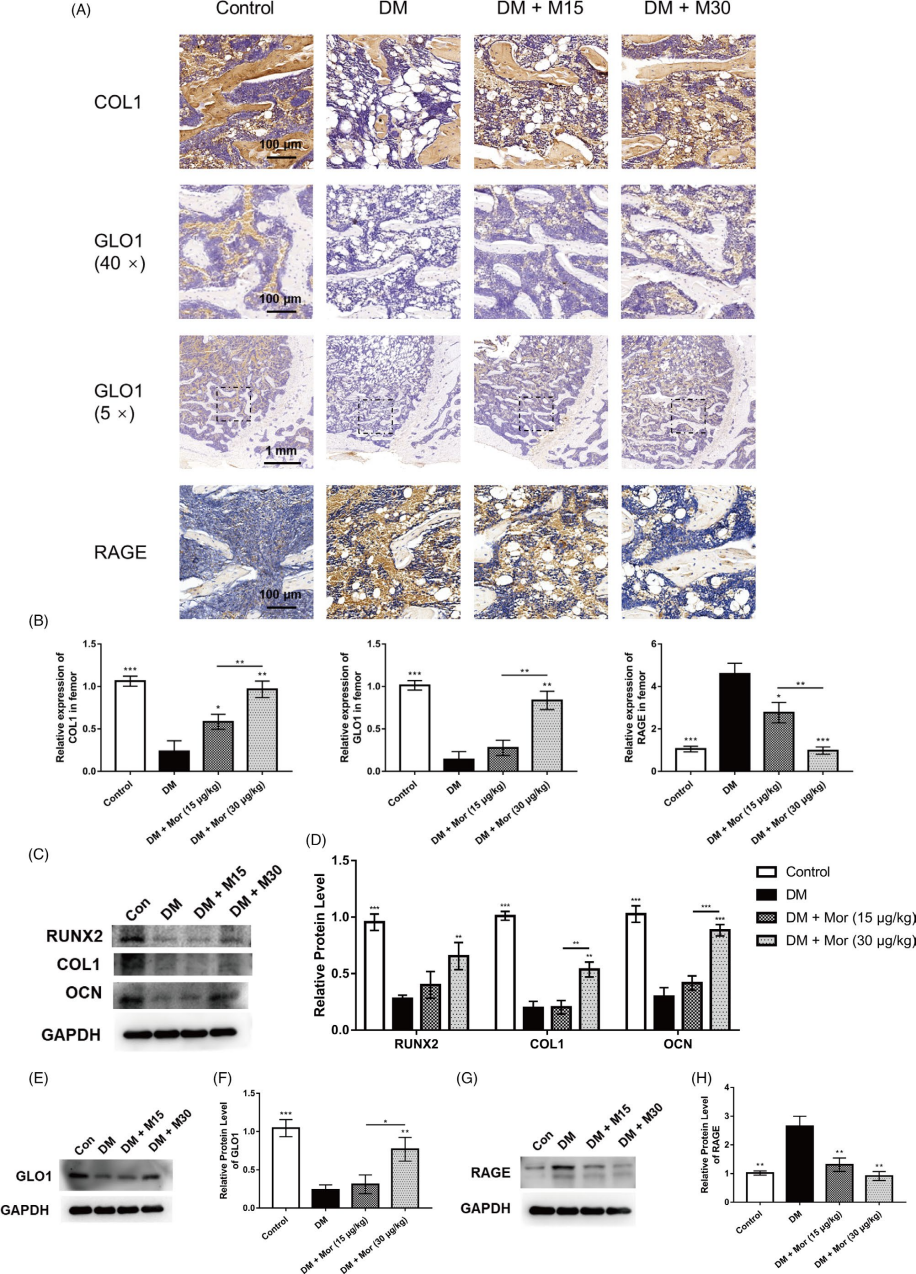
Morroniside restores the expression of RAGE and osteogenic markers to control levels in type 1 diabetes rat model. A and B, Immunohistological analysis of COL1, GLO1 and RAGE in the rat distal femur; magnification at 400 × and 50×. C and D, Expression of osteogenic‐specific proteins were assessed by Western blot. E and F, Expression of GLO1 was measured by Western blot. G and H, Expression of RAGE was measured by Western blot. The data were confirmed by three repeated tests and are presented as the means ± SD. Mor, morroniside. M15, 15 μg/kg morroniside treatment. M30, 30 μg/kg morroniside treatment. *P < .05 compared with the DM group; **P < .01; ***P < .001

To confirm the involvement of AGE‐RAGE and Glo1 in vivo, the protein expression was evaluated in rat distal femurs. The expression levels of osteo‐related proteins, including RUNX2, COL1 and OCN, were suppressed in the DM group compared with the control group and enhanced in the morroniside treatment group (Figure 8C,D). The expression of COL1 as a representative osteo‐specific marker was further confirmed by IHC, which was reduced by DM and attenuated by morroniside administration (Figure 8A). Moreover, IHC and WB results indicated enhanced RAGE and suppressed Glo1 in the DM group compared with the control group. Similar to the effect on BMSCs in vitro, Glo1 expression was significantly enhanced by morroniside treatment, and RAGE expression activated by diabetes was restored to the control levels in the morroniside treatment group (Figure 8A‐H).

## 4 DISCUSSION

**FIGURE 9:**
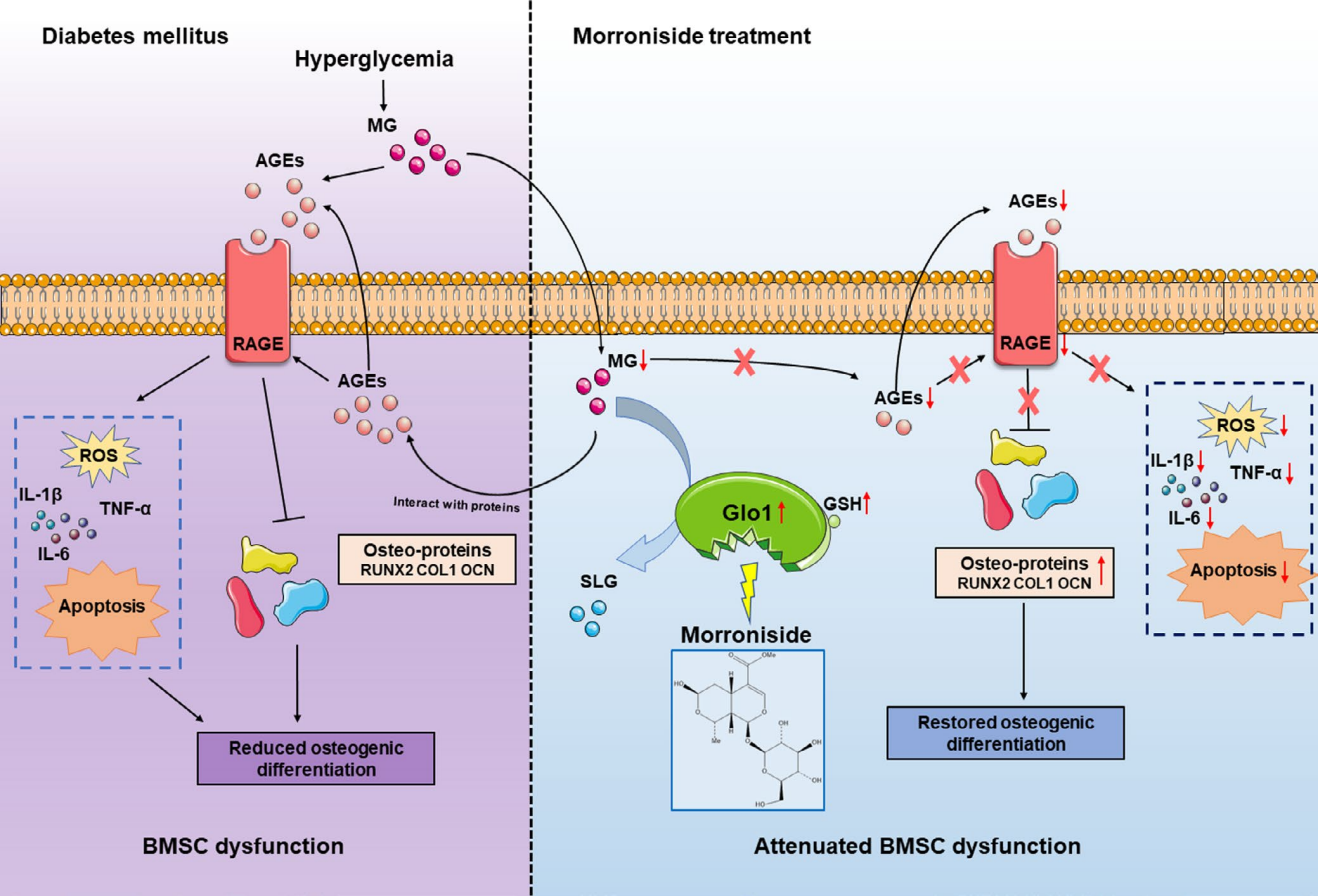
Working model of morroniside attenuation of high glucose–induced BMSC dysfunction. Under pathological diabetic conditions, chronic hyperglycaemia leads to the increased accumulation of AGEs by MG and activation of AGE‐RAGE signalling. Subsequently, AGE‐RAGE activation induces BMSC dysfunction due to a combination of decreased osteogenic differentiation of BMSCs and enhanced inflammation, ROS generation and BMSC apoptosis. Finally, reduced osteogenesis of BMSCs causes impaired bone formation in T1DM. Morroniside triggers Glo1 to degrade MG, thus downregulating AGE‐RAGE signalling, rectifying these downstream detrimental effects and attenuating BMSC dysfunction induced by high glucose. MG, methylglyoxal. AGEs, advanced glycation end products. RAGE, the receptor for advanced glycation end products. ROS, reactive oxygen species. BMSCs, bone marrow mesenchymal stem cells. Glo1, glyoxalase‐1. SLG, S‐d‐lactoylglutathione

In the present study, we found that morroniside relieves high glucose–induced BMSC dysfunction both in vitro and in vivo. In addition, the protective effects of morroniside were at least partly explained by the inhibition of AGE‐RAGE signalling via the activation of Glo1. Although morroniside, the main active component of *C officinalis*, has been used to treat both bone disease and diabetic complications,^17^, ^18^, ^19^ the effect of morroniside on diabetic‐related BMSC dysfunction has not been investigated. Moreover, previous studies have reported that morroniside could regulate RAGE signalling.^24^ Nevertheless, the molecular mechanisms by which morroniside suppresses AGE‐RAGE signalling have not been clearly elucidated. To the best of our knowledge, this is the first study that provides evidence that morroniside can activate Glo1 in bone tissue, which subsequently suppresses AGE‐RAGE signalling. The working model is shown in Figure 9.

Even with insulin replacement therapy, low bone mass and bone fragility are observed in most children and adults with T1DM, and these effects are primarily attributed to impaired bone formation.^29^, ^30^, ^31^ Considerable evidence has shown that BMSCs deteriorate in diabetic conditions and exhibit reduced osteogenic capability.^30^, ^32^ Although the specific mechanism of diabetes‐induced BMSC dysfunction is not fully understood, hyperglycaemia is one of the possible explanations.^4^, ^5^ AGE‐RAGE signalling is pathologically activated by chronic hyperglycaemia and implicated in the pathogenesis of various diabetes‐associated complications.^8^ Previously, RAGE signalling was associated with osteoclastogenesis and bone resorption in the skeleton.^33^, ^34^, ^35^, ^36^ However, more recent studies have increasingly acknowledged that the AGE‐RAGE interaction can also prevent osteogenic differentiation of mesenchymal stem cells, suggesting that RAGE activation may have a detrimental effect on osteogenesis.^12^, ^37^ Thus, attenuating HG‐induced BMSC dysfunction by inhibiting the pathological activation of RAGE signalling could represent a rather promising strategy to prevent impaired bone formation as well as T1DM‐related bone loss.

Glo1 regulates the AGE‐RAGE axis as a key trigger by degrading the important precursor of AGEs (methylglyoxal, MG).^14^ Glutathione (GSH) is a crucial deoxidizer in vivo and is indispensable for MG metabolism as the coenzyme of Glo1; however, it is insufficient under diabetic conditions and leads to decreased Glo1 activity.^15^, ^16^ Recently, a study suggested that antioxidants may exert beneficial effects on Glo1 function, which may be associated with GSH level, and reported that glycine is an antioxidant that increases GSH synthesis and Glo1 activity.^27^ Of note, morroniside has been reported to exhibit antioxidative effects^21^, ^22^; therefore, we hypothesize that morroniside might exert a positive effect on Glo1. Indeed, in the present study, we found that both the expression and activity of Glo1 were increased after morroniside treatment, which was accompanied by the restoration of BMSC functions. Thus, we further concluded that morroniside triggers Glo1 to suppress the HG‐induced pathological activation of AGE‐RAGE signalling. Nevertheless, the precise mechanism underlying the role of morroniside in activating Glo1 and enhancing GSH synthesis remains unclear. Several studies reported that nuclear factor erythroid‐2‐related factor 2 (Nrf2) and antioxidant response elements (AREs) are associated with the regulation of Glo1 expression,^38^ thus providing possible directions for further investigation.

Meanwhile, enhanced apoptosis, oxidative stress and inflammation also play an unignorable role in T1DM‐related BMSC dysfunction. Under chronic HG conditions, AGEs and RAGE activation could promote the apoptosis of BMSCs via caspase activation, which involves TNF‐α production and oxidative stress.^39^, ^40^ Furthermore, increased inflammation and oxidative stress both exhibit detrimental effects on the osteogenic differentiation of BMSCs because the prolonged expression of TNF‐α and greater production of ROS reduce the expression of factors that are crucial for BMSC osteogenic differentiation.^41^, ^42^ However, the relationships between apoptosis and osteogenic differentiation in BMSCs remain unclear. One opinion is that apoptosis could upstream regulate the osteogenic differentiation of BMSCs. Although direct evidence has not been observed, some studies have shown that apoptosis could regulate differentiation in megakaryocytes.^43^ Therefore, we infer that the downregulation of apoptosis by morroniside may possibly promote osteogenic differentiation in BMSCs. Another opinion is that apoptosis does not regulate osteogenic differentiation in BMSCs. Some studies have shown that apoptosis and osteogenic differentiation of BMSCs could be regulated by one upstream factor, such as RAGE.^35^ In other words, apoptosis and osteogenic differentiation might be regulated by AGE‐RAGE signalling independently. In brief, the direct effect of apoptosis on the osteogenic differentiation of BMSCs remains unclear and needs further exploration.

Morroniside has several advantages for future potential use. First, morroniside has favourable biocompatibility. Morroniside is the main active component of *C officinalis*, which is an official drug listed in the *Chinese Pharmacopoeia* and has been used for more than two thousand years.^44^ In addition, morroniside shows no toxic effects in various cell types and has no obvious damaging effects in animals.^20^, ^21^, ^22^, ^23^, ^45^, ^46^, ^47^ Second, morroniside is economically efficient. *C officinalis* has a large volume of production, and the separation and purification techniques have been well established. Thus, the cost of morroniside is relatively low, which makes it affordable for patients in less developed countries. Third, as an iridoid glycoside, morroniside is stable and soluble and can be easily transported and preserved. Consequently, morroniside could be a prospective candidate for future clinical use.

The present study has several limitations. First, the detailed mechanisms underlying the ability of morroniside to activate Glo1 and the osteogenic effects of morroniside on inhibiting AGE‐RAGE signalling have not been fully elucidated. Second, pharmacological parameters such as the optimal drug doses and drug treatment timing that are critical for clinical translational studies have not been well explored. Third, BMSC osteogenic differentiation in vivo was only determined via WB analysis. Future work should target the molecular mechanisms underlying the protective effect of morroniside and explore its optimal administration in large animal models.

In conclusion, the present study demonstrates that morroniside attenuates high glucose–induced BMSC dysfunction partly via the inhibition of AGE‐RAGE signalling through the activation of Glo1. The use of morroniside may provide a new strategy that targets T1DM‐related impaired bone formation and has therapeutic potential for the treatment of diabetic osteoporosis.

## CONFLICTS OF INTEREST

The authors declare no conflicts of interest.

## Supporting information

Fig S1

## Data Availability

The data that support the findings of this study are available from the corresponding author upon reasonable request.
