# Supplementary figures and images for: Morroniside attenuates high glucose–induced BMSC dysfunction by regulating the Glo1/AGE/RAGE axis
Source: Cell Prolif. 2020 Jul 9;53(8):e12866. doi: 10.1111/cpr.12866 (PMC7445400; doi:10.1111/cpr.12866)

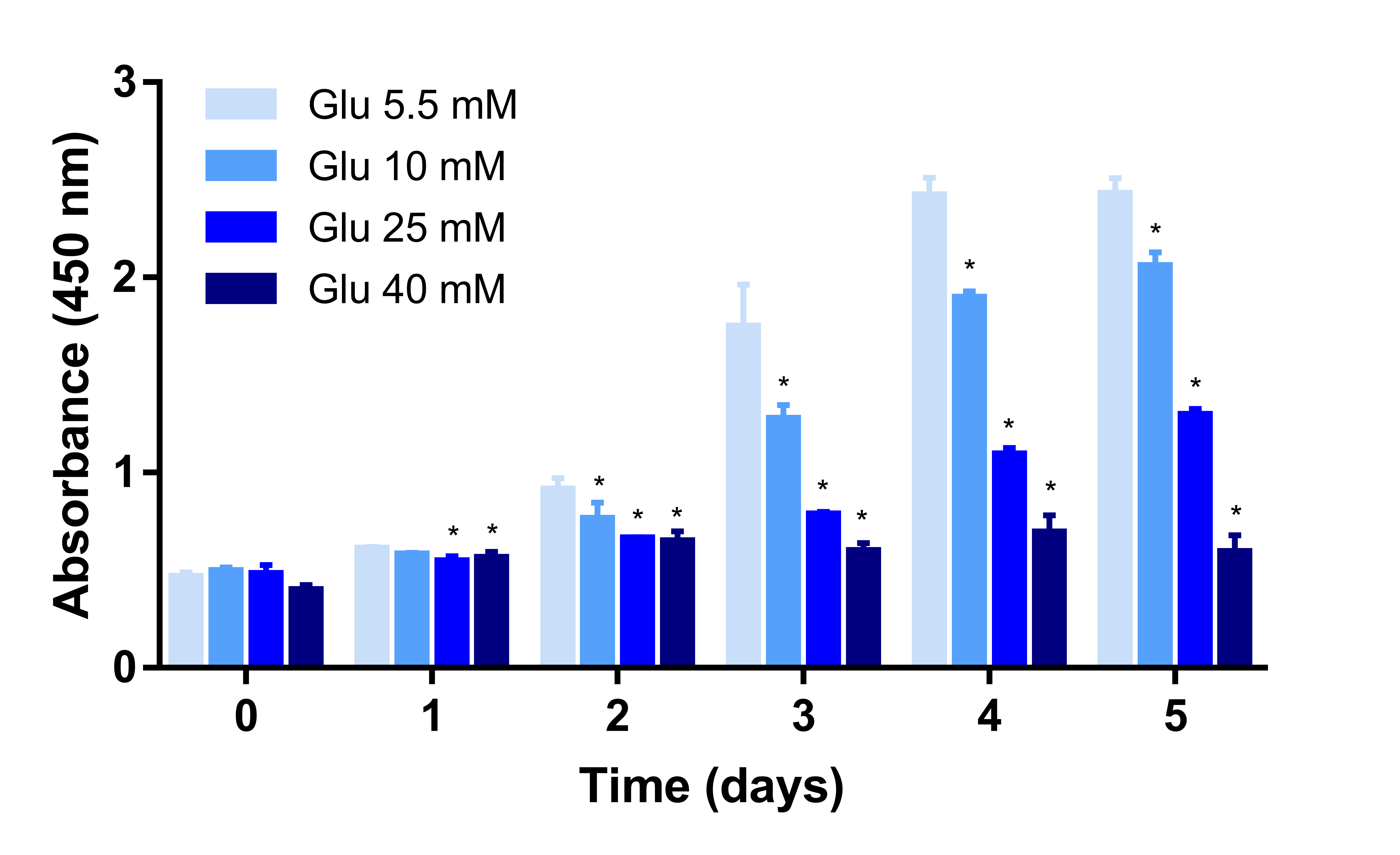

Supplement: Supplementary file 1 — Fig S1 [file CPR-53-e12866-s001.tif]
